# Carbon Isotope Fractionation during the Formation of CO_2_ Hydrate and Equilibrium Pressures of ^12^CO_2_ and ^13^CO_2_ Hydrates

**DOI:** 10.3390/molecules26144215

**Published:** 2021-07-11

**Authors:** Hiromi Kimura, Go Fuseya, Satoshi Takeya, Akihiro Hachikubo

**Affiliations:** 1 Kitami Institute of Technology, Graduate School of Engineering, 165 Koen-cho, Kitami 090-8507, Japan; mayu011970@gmail.com (H.K.); fuseya1992@gmail.com (G.F.); 2 National Metrology Institute of Japan (NMIJ), National Institute of Advanced Industrial Science and Technology (AIST), Central 5, Higashi 1-1-1, Tsukuba 305-8565, Japan; s.takeya@aist.go.jp; 3 Environmental and Energy Resources Research Center, Kitami Institute of Technology, 165 Koen-cho, Kitami 090-8507, Japan

**Keywords:** CO_2_ hydrate, carbon isotope, isotopic fractionation, phase equilibrium, Raman spectra

## Abstract

Knowledge of carbon isotope fractionation is needed in order to discuss the formation and dissociation of naturally occurring CO_2_ hydrates. We investigated carbon isotope fractionation during CO_2_ hydrate formation and measured the three-phase equilibria of ^12^CO_2_–H_2_O and ^13^CO_2_–H_2_O systems. From a crystal structure viewpoint, the difference in the Raman spectra of hydrate-bound ^12^CO_2_ and ^13^CO_2_ was revealed, although their unit cell size was similar. The δ^13^C of hydrate-bound CO_2_ was lower than that of the residual CO_2_ (1.0–1.5‰) in a formation temperature ranging between 226 K and 278 K. The results show that the small difference between equilibrium pressures of ~0.01 MPa in ^12^CO_2_ and ^13^CO_2_ hydrates causes carbon isotope fractionation of ~1‰. However, the difference between equilibrium pressures in the ^12^CO_2_–H_2_O and ^13^CO_2_–H_2_O systems was smaller than the standard uncertainties of measurement; more accurate pressure measurement is required for quantitative discussion.

## 1. Introduction

Gas hydrates are crystalline clathrate compounds that have guest gas molecules encapsulated in hydrogen-bonded water cages and can be thermodynamically stable at high pressures and low temperatures. The phase equilibrium pressure-temperature conditions vary depending on the guest molecule type [1]. Gas hydrates with encapsulated natural gases exist in sub-marine/sublacustrine sediments and below the permafrost on Earth. Because they contain copious amounts of greenhouse gases, such as methane, concerns arose that their dissociation could add to global warming [2,3,4]. Hydrate-bound gas contains both hydrocarbons and CO_2_ [5,6,7], and natural gas hydrates encapsulating CO_2_ have been observed at the venting sites of liquid CO_2_ [8,9]. On the other hand, the possibility of existing CO_2_ hydrates on Mars was proposed [10,11] and the conditions for CO_2_ hydrate formation on Mars have been discussed [12]. As above, CO_2_ hydrates are critical in both geochemistry and space science and require better understanding. 

Since CO_2_ comprises carbon and oxygen atoms, several isotopic species of CO_2_ exist, depending on the combination of stable isotopes (isotopologues: ^12^C, ^13^C, ^16^O, ^17^O, and ^18^O). For example, the abundance ratio of ^13^CO_2_ is ~1.1% of the total CO_2_ and the rest is almost ^12^CO_2_. Stable isotope fractionation of the guest gas during the gas hydrate formation provides information for discussing the formation, maintenance, and decomposition processes of gas hydrates. For example, the trend of hydrogen and carbon isotope fractionation during the formation of synthetic methane and ethane hydrates was investigated [13]. Moreover, the results were applied to estimate the formation process of natural gas hydrates [14,15,16]. Carbon isotope fractionation during the formation of CO_2_ hydrates was reported [17], revealing that the CO_2_ δ^13^C in the hydrate phase was 0.9‰ lower than that in the gas phase at 268 K [17]. Therefore, ^12^CO_2_ is more easily encapsulated in the hydrate phase than ^13^CO_2_. Because the temperature of existing natural gas hydrates at sea/lake bottom sediments is above 273 K, information about carbon isotope fractionation above the freezing point of water is needed to discuss the formation and dissociation of naturally occurring CO_2_ hydrates. Furthermore, CO_2_ hydrates have been suggested outside of Earth. Therefore, it is also necessary to confirm isotope fractionation in a wider temperature range.

In studies on methane hydrates, the equilibrium pressures of CH_3_D and CD_4_ hydrates were ~0.04 MPa and ~0.14 MPa higher than those of the CH_4_ hydrate, respectively [18]. Because guest molecules, which have lower equilibrium pressure, are preferentially encapsulated in the gas hydrate cages, the difference in equilibrium pressures of CH_3_D and CH_4_ hydrates can explain the hydrogen isotope fractionation in methane during methane hydrate formation [18]. Thus, comparing the phase equilibrium *p*–*T* conditions of each gas hydrate encapsulating isotopologues can explain the trend of isotopic fractionation of guest gases during gas hydrate formation. However, the equilibrium pressure of ^13^CO_2_ hydrates has not been reported.

In this study, we synthesized gas hydrate samples, encapsulated CO_2_ isotopologues (^12^CO_2_ and ^13^CO_2_), and characterized their crystallographic properties using powder X-ray diffraction (PXRD) and Raman spectroscopy. We also measured the equilibrium pressures of ^12^CO_2_ and ^13^CO_2_ hydrates at a temperature range between 269 K and 278 K. We investigated carbon isotope fractionation between hydrate-bound gas and residual gas in a pressure cell in the temperature range of 226 K to 278 K.

## 2. Results and Discussion

We confirmed the crystallographic structures of ^12^CO_2_ and ^13^CO_2_ hydrates and obtained their lattice constants using the PXRD method. The diffraction patterns of cubic structure I (sI) hydrates were observed from these hydrates (Figure S1). The lattice constants of ^12^CO_2_ and ^13^CO_2_ hydrates were similar at 11.8352(6) Å and 11.8323(5) Å, respectively.

**Figure 1:**
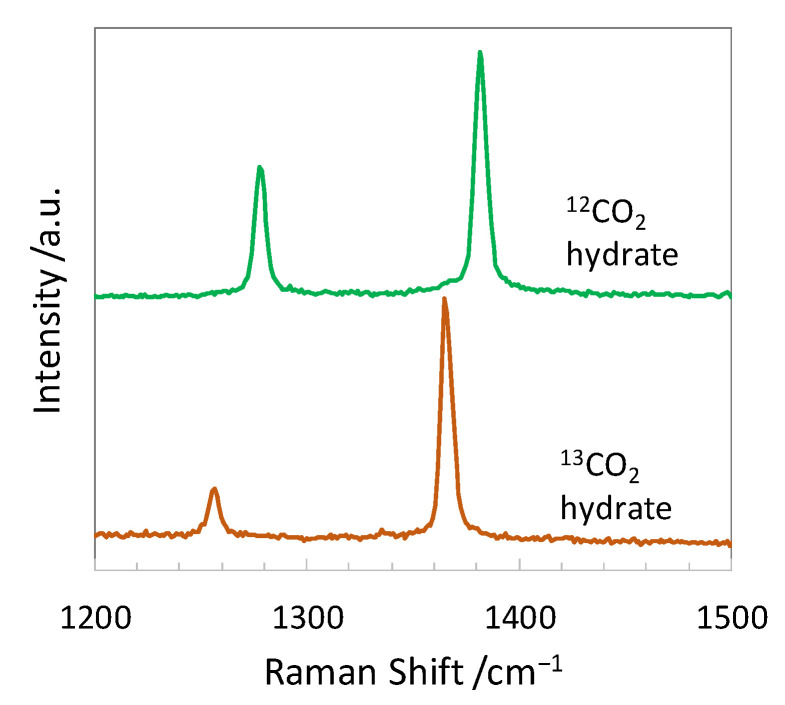
Raman spectra of ^12^CO_2_ and ^13^CO_2_ hydrates in the CO stretching vibration mode region of CO_2_. The spectra were recorded at atmospheric pressure and 140 K. (a.u., arbitrary units).

Figure 1 shows the Raman spectra of ^12^CO_2_ and ^13^CO_2_ hydrates and Table 1 summarizes the observed Raman shifts of hydrate-bound isotopologue CO_2_ and their assignments to the vibrational modes. Two distinct peaks corresponding to the Fermi dyad of CO_2_ in the hydrate cages were observed at 1278.2 cm^−1^ and 1381.9 cm^−1^ for ^12^CO_2_ hydrates, corresponding to [19,20,21]. These peaks shifted to 1256.4 cm^−1^ and 1365.8 cm^−1^ for the ^13^CO_2_ hydrate. Qin and Kuhs [21] observed 1366.6 ± 4.0 cm^−1^ for the upper Fermi dyad of hydrate-bound ^13^CO_2_, and our data were consistent with their results. Definite differences (22 cm^−1^ and 16 cm^−1^ for lower and upper peaks, respectively) were found between these Raman peaks caused by the encapsulated ^12^CO_2_ and ^13^CO_2_ molecules.

**Figure 2:**
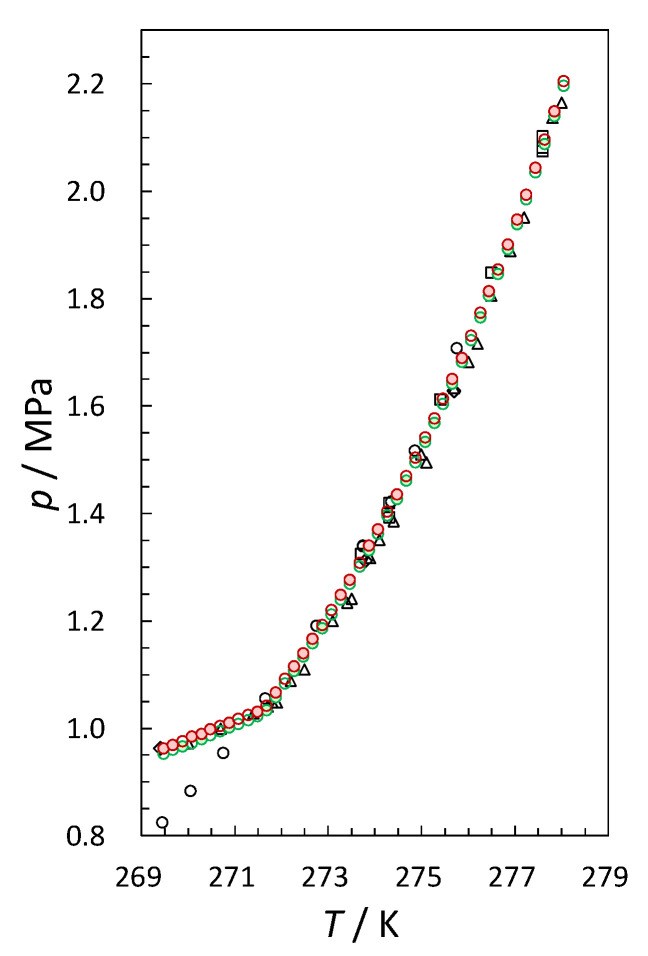
Three-phase (ice/water + hydrate + vapor) equilibrium *p*–*T* conditions for ^12^CO_2_ and ^13^CO_2_ hydrates. Green circles, ^12^CO_2_–H_2_O (this work); brown circles, ^13^CO_2_–H_2_O (this work); open squares, ^12^CO_2_–H_2_O [24]; open triangles, ^12^CO_2_–H_2_O [25]; open diamonds, ^12^CO_2_–H_2_O [26]; open circles, ^12^CO_2_–H_2_O [23].

Table 2 lists and Figure 2 plots the *p*–*T* data for the three-phase equilibrium (ice/water + hydrate + vapor) for the ^12^CO_2_–H_2_O and ^13^CO_2_–H_2_O systems. The phase in the equilibrium of each point in Table 2 was determined using the quadruple point of the ^12^CO_2_–H_2_O system (1.04 MPa and 271.6 K) reported by [23]. To ensure the accuracy of the experimental apparatus described in the previous section, we evaluated the equilibrium *p*–*T* data of ^12^CO_2_ hydrates. From Figure 2, they correlate well with the literature [23,24,25,26]. The equilibrium pressures in the ^13^CO_2_–H_2_O system are higher by 0.007–0.012 MPa compared with the corresponding values in the ^12^CO_2_–H_2_O system between 269 K and 278 K. However, these differences are within the range of uncertainty of the pressure measurements.

**Figure 3:**
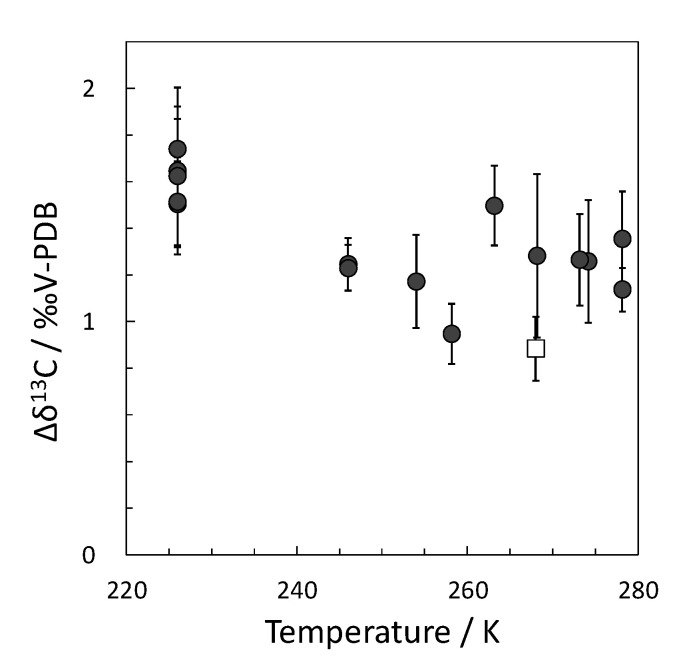
Δδ^13^C at the formation of CO_2_ hydrates that formed in the temperature range of 226–278 K. (Solid circles, this work; open square, literature) [17].

Figure 3 shows the differences in δ^13^C between the residual and hydrate-bound CO_2_ (Δδ^13^C) in the temperature range of 226 K to 278 K. The δ^13^C of hydrate-bound CO_2_ was lower (1.0–1.5‰) than that of residual CO_2_ in the temperature range used in this study. This result correlates with a previous study that reported ~0.9‰ of Δδ^13^C at 268 K [17]. These results indicate that ^12^CO_2_ molecules are preferentially encapsulated in hydrate cages as guest molecules rather than ^13^CO_2_ molecules during the formation of CO_2_ hydrates in the temperature range of 226 K to 278 K.

An earlier study reported hydrogen isotope fractionation in methane during the formation of methane hydrates [13]. The δD of hydrate-bound methane was 4.8 ± 0.4‰ lower than that of residual molecules [13]. On the other hand, the equilibrium pressures of CH_3_D and CD_4_ hydrates were ~0.04 MPa and ~0.14 MPa higher than those of the CH_4_ hydrate, respectively [18]. For example, the equilibrium pressure of the C_2_H_6_ hydrate is lower than that of the CH_4_ hydrate [24], resulting in a preferential C_2_H_6_ concentration in the hydrate phase during the formation process of CH_4_ and C_2_H_6_ mixed-gas hydrate [24,27]. Ozeki et al. explained that the difference in equilibrium pressures between CH_3_D and CH_4_ hydrates causes the isotopic fractionation of hydrogen in methane during the formation of methane hydrates [18]. In this study, carbon isotope fractionation in CO_2_ (the difference in δ^13^C between the residual and hydrate gases) during the formation of CO_2_ hydrates was 1.0–1.5‰ (Figure 3). As above, this trend of carbon isotope fractionation of CO_2_ is reasonable because the equilibrium pressures of the ^12^CO_2_–H_2_O system seem slightly lower than those of the ^13^CO_2_–H_2_O system. However, it cannot be discussed here because these differences in equilibrium pressures (Table 2) are smaller than the experimental uncertainty (0.05 MPa).

## 3. Materials and Methods

### 3.1. Crystallographic Analysis

We formed fine powder samples of ^12^CO_2_ and ^13^CO_2_ hydrates in small pressure cells (internal volume: 8 mL). Research-grade CO_2_ (purity 99.999% for CO_2_, including ~1.1% of ^13^CO_2_, Takachiho Chemical Industrial) and ^13^CO_2_ (purity 99%, Taiyo Nippon Sanso) were used as the guest ^12^CO_2_ and ^13^CO_2_, respectively. 1 g of fine ice powder was placed in the high-pressure cell, and the air was vacuumed at 77 K. CO_2_ isotopologues were introduced to each cell and the temperature was increased from 77 K to 273.2 K to form their gas hydrates. CO_2_ sublimated and increased the internal pressure. We confirmed hydrate formation by the decrease in pressure at 273.2 K. The samples were recovered and stored at 77 K for the crystallographic analysis.

PXRD measurements were performed using an X-ray diffractometer (model Ultima-III, Rigaku Co., Tokyo, Japan) with parallel beam optics and a low-temperature chamber. Finely-powdered hydrate samples were mounted on a PXRD sample holder made of 2.5 mm thick Cu at 93 K. Each measurement was performed in a *θ*/2*θ* step scan mode with a step width of 0.02° using Cu Kα radiation (λ = 1.541 Å).

We obtained the Raman spectra of ^12^CO_2_ and ^13^CO_2_ hydrates. A Raman spectrometer (RMP-210, Jasco Co., Tokyo, Japan) was used equipped with a 532 nm excitation source (100 mW), a single holographic diffraction grating (1800 grooves per mm), and a charged coupled device detector. The spectrum pixel resolution, which is the spectrum’s sampling interval, was 1.1 cm^−1^ per pixel in the range of 1200–1400 cm^−1^. The wavenumber was calibrated using atomic emission lines from a neon lamp. The Raman spectra for the C–O symmetric stretch region (1200–1400 cm^−1^) of the encapsulated CO_2_ molecules in the gas hydrate water cages were obtained at ambient pressure and 140 K using a cooling stage (THMS600, Linkam Scientific Instruments Ltd., Tadworth, UK). The peak positions could be rigorously analyzed by fitting the data to a Voigt function, allowing us to obtain high positional accuracy.

### 3.2. Measurement for Equilibrium Pressure

To obtain the data of equilibrium pressures of ^12^CO_2_ and ^13^CO_2_ hydrates, we formed them in the same small pressure cells as those used for crystallographic analysis. The experimental setup to achieve their equilibrium condition was described in [18]. 1 g of fine ice powder was placed in the cell, evacuated at 77 K, and CO_2_ isotopologues were introduced in the amount needed to achieve equilibrium conditions of the ice/liquid water, hydrates, and vapor at ~273.2 K. The hydrate-enclathrated CO_2_ isotopologues were formed by melting the ice powder at 273.2 K under high pressure of CO_2_. The decrease in pressure due to hydrate formation was observed at 273.2 K.

The equilibrium hydrate dissociation and formation conditions were determined using a phased isochoric method of heating and cooling [18]. Three-phase (ice/water + hydrate + vapor) equilibrium conditions were achieved by increasing the temperature by 0.4 K and then decreasing it by 0.2 K. Since two values of equilibrium pressure at each temperature by heating and cooling were obtained, we determined the phase equilibrium points as their average temperatures and pressures. The phase equilibrium data of ^12^CO_2_ and ^13^CO_2_ hydrates were obtained between 269 K and 278 K. The uncertainties of the temperature and pressure measurements were 0.05 K and 0.05 MPa, respectively.

### 3.3. Gas Analysis for Detecting Carbon Isotope Fractionation

The preparation method of gas hydrate samples for measuring isotopic fractionation was the same as that of [13]. Research-grade CO_2_ gas was used as the guest gas (purity 99.999% for CO_2_, including ~1.1% of ^13^CO_2_, Takachiho Chemical Industrial). Distilled and deionized water was used as host molecules. The temperature effect on isotope fractionation was confirmed by forming CO_2_ hydrate samples at 226 K, 246 K, 254 K, 258 K, 263 K, 268 K, 273 K, 274 K, and 278 K. For the samples formed below the freezing point of water, fine ice powder (0.7 g) was filled in a high-pressure cell (internal volume: 42 mL) in a cold room at 253 K. For the samples formed above the freezing point of water, 5 g water was filled into a high-pressure cell equipped with a stirring device (internal volume: 150 mL). These high-pressure cells were cooled to below 90 K, vacuumed inside the air, and CO_2_ was introduced into the cell. The amount of CO_2_ was controlled to reach above the equilibrium pressure of CO_2_ hydrates and below the CO_2_ liquefaction pressure at each temperature. These cells were set into a circulating constant-temperature bath (>255 K) or cold rooms (226 K and 246 K) to maintain each temperature for hydrate formation. The trapped CO_2_ in the cells sublimated and reached the desired pressure at each temperature. The internal pressure decreased as the CO_2_ hydrates formed. When the pressure stabilized and the pressure decrease rate was lower than 0.01 MPa h^−1^, the residual gas that was not encapsulated in the gas hydrate was collected. The cell was cooled below 90 K and the hydrate sample was recovered from the cell. The residual and hydrate-bound gases were retrieved in a vacuum line system and their pressures were adjusted to atmospheric pressure.

Each gas sample was introduced into a continuous-flow isotope ratio mass spectrometer (CF-IRMS, Delta V, Thermo Fisher Scientific Inc., Waltham, MA, USA) coupled with a gas chromatograph (TRACE GC Ultra, Thermo Fisher Scientific Inc., Waltham, MA, USA) using a syringe injection. The gas chromatograph was equipped with a CP-PoraPLOT Q capillary column (length 25 m, ID 0.32 mm, film thickness 10 μm, Agilent Technologies). Carbon isotope compositions were reported as δ values (‰),

(1) $$\delta \;\left[‰\right]=\left(\frac{R_{sample}-R_{standard}}{R_{standard}}\right)\times 1000$$

where *R* denotes the ^13^C/^12^C ratio. δ^13^C is given referring to the V-PDB standards, determined using NIST RM8544 (NBS19). The analytical precision was 0.1‰. The difference between the δ^13^C of the hydrate-bound gas and that of the residual gas was determined (δ^13^C of the residual gas − δ^13^C of the hydrate-bound gas, defined as Δδ^13^C).

## 4. Conclusions

We synthesized isotopologue CO_2_ hydrates to obtain their crystallographic properties. From the Raman spectra of ^12^CO_2_ and ^13^CO_2_ hydrates, definite differences were found in the Raman shift of Fermi dyad of ^12^CO_2_ and ^13^CO_2_ encapsulated in hydrate cages, although their unit cell size was similar. We investigated carbon isotope fractionation during the formation of CO_2_ hydrates and measured the three-phase equilibria of ^12^CO_2_–H_2_O and ^13^CO_2_–H_2_O systems. The δ^13^C of hydrate-bound CO_2_ was lower than that of the residual CO_2_ (1.0–1.5‰) in the formation temperature range between 226 K to 278 K. From the results of isotopic fractionation, differences in equilibrium pressures were expected. The equilibrium pressures in the ^12^CO_2_–H_2_O system were slightly lower by 0.007–0.012 MPa compared with the corresponding values in the ^13^CO_2_–H_2_O system between 269 K to 278 K. We concluded that the small difference in equilibrium pressures of ~0.01 MPa between ^12^CO_2_ and ^13^CO_2_ hydrates causes carbon isotope fractionation of 1.0~1.5‰. However, the pressure differences obtained were within the range of the uncertainty of the pressure measurements. More accurate measurements of equilibrium pressure are needed for further discussion. These results caused by carbon isotope fractionation will be useful for a better understanding of the formation and dissociation of naturally occurring CO_2_ hydrates.

## Figures and Tables

**Table 1:** Observed Raman shifts of hydrate-bound CO_2_ and assignments to the vibrational modes.

Guest Molecule	Raman Shift Gas/cm^−1^	Raman Shift Hydrate/cm^−1^	Assign	Vibrational Mode
^12^CO_2_	1285.40 [22]	1278.2 ^a^, 1277 [19], 1278 [20], 1275.5 ± 0.8 [21]	υ_1_ + 2υ_2_^b^	CO s-stretch + bend ^b^
	1388.15 [22]	1381.9 ^a^, 1381 [19], 1382 [20], 1379.4 ± 0.8 [21]		
^13^CO_2_	1266.03 [22]	1256.4 ^a^		
	1369.90 [22]	1365.8 ^a^, 1366.6 ± 4.0 [21]		

^a^ This work, uncertainty is <1.1 cm^−1^. ^b^ Fermi resonance.

**Table 2:** Three-phase equilibrium *p*–*T* conditions in ^12^CO_2_–H_2_O and ^13^CO_2_–H_2_O systems ^a^.

*T*/K	*p*_12CO2_–H_2_O/MPa	*p*_13CO2_–H_2_O/MPa	Phase in Equilibrium
269.47	0.953	0.963	ice + hydrate + vapor
269.67	0.960	0.969	ice + hydrate + vapor
269.88	0.967	0.977	ice + hydrate + vapor
270.08	0.973	0.984	ice + hydrate + vapor
270.28	0.980	0.990	ice + hydrate + vapor
270.47	0.987	0.999	ice + hydrate + vapor
270.67	0.994	1.004	ice + hydrate + vapor
270.88	1.002	1.011	ice + hydrate + vapor
271.08	1.009	1.018	ice + hydrate + vapor
271.28	1.016	1.025	ice + hydrate + vapor
271.48	1.023	1.031	ice + hydrate + vapor
271.67	1.034	1.043	water + hydrate + vapor
271.88	1.058	1.067	water + hydrate + vapor
272.08	1.083	1.092	water + hydrate + vapor
272.28	1.109	1.118	water + hydrate + vapor
272.46	1.133	1.142	water + hydrate + vapor
272.66	1.159	1.168	water + hydrate + vapor
272.86	1.185	1.195	water + hydrate + vapor
273.05	1.212	1.223	water + hydrate + vapor
273.25	1.240	1.251	water + hydrate + vapor
273.45	1.270	1.280	water + hydrate + vapor
273.66	1.302	1.312	water + hydrate + vapor
273.85	1.333	1.342	water + hydrate + vapor
274.05	1.365	1.376	water + hydrate + vapor
274.26	1.397	1.408	water + hydrate + vapor
274.47	1.427	1.435	water + hydrate + vapor
274.67	1.461	1.469	water + hydrate + vapor
274.87	1.496	1.504	water + hydrate + vapor
275.07	1.534	1.542	water + hydrate + vapor
275.27	1.569	1.578	water + hydrate + vapor
275.46	1.605	1.614	water + hydrate + vapor
275.66	1.642	1.651	water + hydrate + vapor
275.86	1.682	1.690	water + hydrate + vapor
276.06	1.723	1.732	water + hydrate + vapor
276.26	1.766	1.774	water + hydrate + vapor
276.44	1.806	1.814	water + hydrate + vapor
276.64	1.846	1.855	water + hydrate + vapor
276.85	1.893	1.901	water + hydrate + vapor
277.04	1.939	1.948	water + hydrate + vapor
277.24	1.985	1.994	water + hydrate + vapor
277.43	2.035	2.044	water + hydrate + vapor
277.64	2.088	2.097	water + hydrate + vapor
277.84	2.140	2.149	water + hydrate + vapor
278.05	2.196	2.205	water + hydrate + vapor

^a^ Uncertainties of *T* and *p* are 0.05 K and 0.05 MPa, respectively.

## Data Availability

Available from the authors.

## Supplementary Materials

The following is available online: Figure S1, Powder X-ray diffraction patterns of the hydrate enclathrated CO_2_ isotopologues.

## Sample Availability

Samples of the compounds are not available from the authors.
