# Supplementary material for: Carbon Isotope Fractionation during the Formation of CO2 Hydrate and Equilibrium Pressures of 12CO2 and 13CO2 Hydrates
Source: Molecules. 2021 Jul 11;26(14):4215. doi: 10.3390/molecules26144215 (PMC8306263; doi:10.3390/molecules26144215)

Supplementary Materials

Carbon Isotope Fractionation during the Formation of CO_2 Hydrate and Equilibrium Pressures of $^{12}CO_2$ and $^{13}CO_2$ Hydrates

Hiromi Kimura [1], Go Fuseya [1], Satoshi Takeya [2] and Akihiro Hachikubo [3,*]

[1] Graduate School of Engineering, Kitami Institute of Technology, 165 Koen-cho, Kitami 090-8507, Japan; mayu011970@gmail.com (H.K.); fuseya1992@gmail.com (G.F.)
[2] National Metrology Institute of Japan (NMIJ), National Institute of Advanced Industrial Science and Technology (AIST), Central 5, Higashi 1-1-1, Tsukuba 305-8565, Japan; s.takeya@aist.go.jp
[3] Environmental and Energy Resources Research Center, Kitami Institute of Technology, 165 Koen-cho, Kitami 090-8507, Japan
* Correspondence: hachi@mail.kitami-it.ac.jp

*Figure S1. Powder X-ray diffraction patterns of the mixture of sI hydrate enclathrated CO_2 isotopologues and hexagonal ice Ih. (a) $^{12}CO_2$ hydrate; (b) $^{13}CO_2$ hydrate.

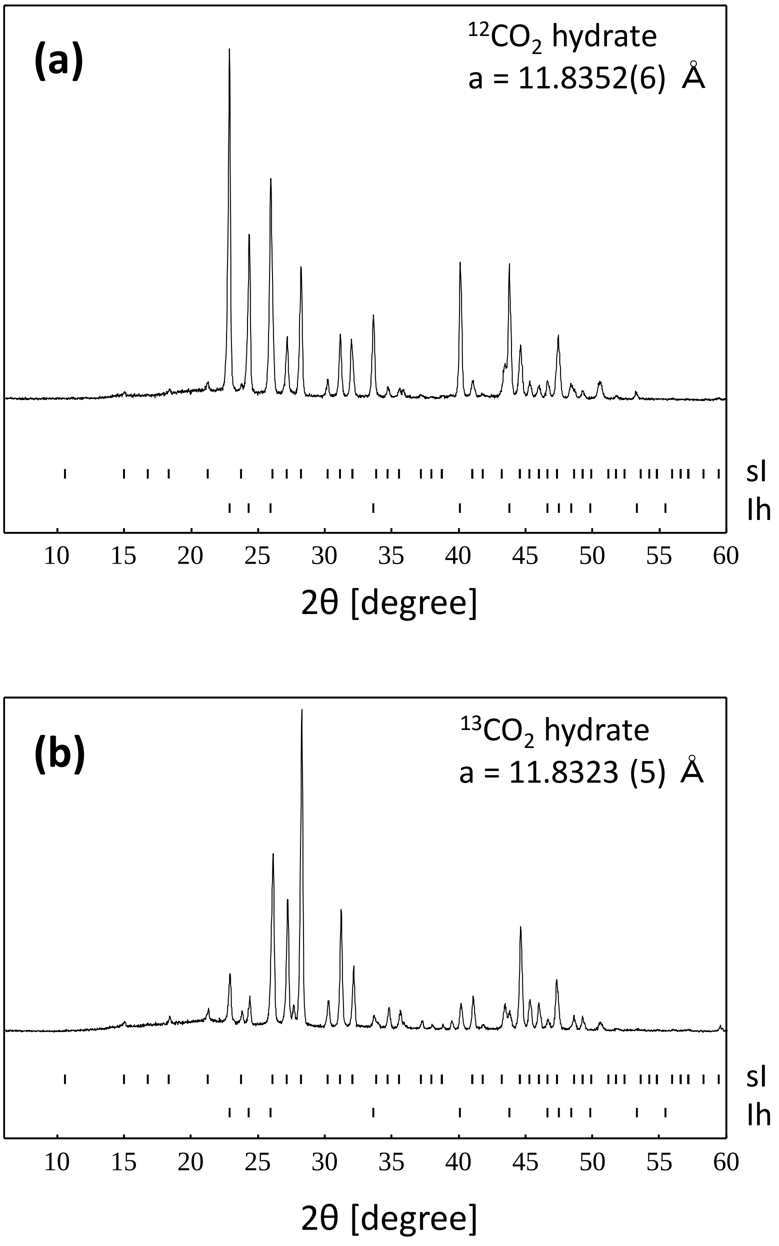

Supplement: Supplementary file 1 [file molecules-26-04215-s001.zip › molecules-1276539-supplementary.pdf]
